# Ferroelectric Electroresistance after a Breakdown in Epitaxial Hf_0.5_Zr_0.5_O_2_ Tunnel Junctions

**DOI:** 10.1021/acsaelm.2c01186

**Published:** 2023-01-30

**Authors:** Xiao Long, Huan Tan, Florencio Sánchez, Ignasi Fina, Josep Fontcuberta

**Affiliations:** Institut de Ciència de Materials de Barcelona (ICMAB-CSIC), Campus UAB, Bellaterra08193, Catalonia, Spain

**Keywords:** ferroelectric, ferroelectric hafnium oxide, epitaxial HfO_2_, ferroelectric tunnel junction, resistive switching

## Abstract

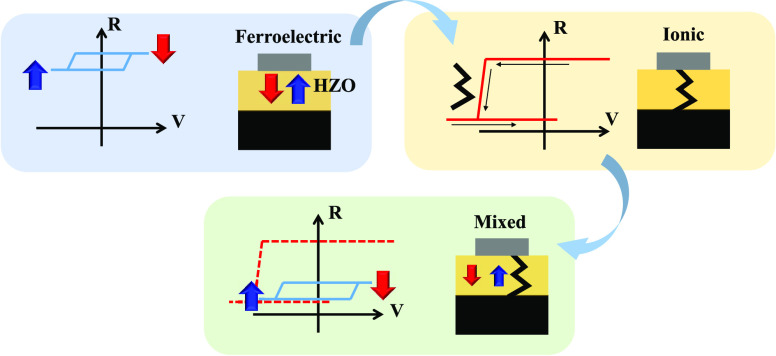

The recent discovery of ferroelectricity in doped HfO_2_ has opened perspectives on the development of memristors based on ferroelectric switching, including ferroelectric tunnel junctions. In these devices, conductive channels are formed in a similar manner to junctions based on nonferroelectric oxides. The formation of the conductive channels does not preclude the presence of ferroelectric switching, but little is known about the device ferroelectric properties after conduction path formation or their impact on the electric modulation of the resistance state. Here, we show that ferroelectricity and related sizable electroresistance are observed in pristine 4.6 nm epitaxial Hf_0.5_Zr_0.5_O_2_ (HZO) tunnel junctions grown on Si. After a soft breakdown induced by the application of suitable voltage, the resistance decreases by about five orders of magnitude, but signatures of ferroelectricity and electroresistance are still observed. Impedance spectroscopy allows us to conclude that the effective ferroelectric device area after the breakdown is reduced, most likely by the formation of conducting paths at the edge.

## Introduction

New paradigms in computing architectures, such as neuromorphic computing, require materials with the ability to show memristive responses with reduced power consumption and high reliability.^1,2^ The discovery of ferroelectricity in doped hafnium oxide has enabled the development of high-density memory devices based on ferroelectric polarization switching because hafnium oxide is fully compatible with complementary metal–oxide–semiconductor (CMOS) technology.^3−5^ In particular, ferroelectric tunnel junctions based on hafnium oxide, where the tunneling current and junction resistance are modulated by ferroelectric polarization and concomitantly by the suitable application of an applied electric field (electroresistance, denoted as ER), are of potential interest for applications. Thus, the ER phenomenon has been widely studied in ferroelectric HfO_2_ devices.^6−20^ Besides, nonferroelectric HfO_2_ has been employed since long ago as a high-k material in electronics and is a prime candidate for data storage as it also displays a large change of ER by suitable voltage control. In the latter case, the resistance changes (resistivity switching) are ascribed to ionic motion, for instance, the formation of a conductive filament or other types of conductive regions usually formed at the grain boundaries.^21−27^ In general, ER values associated with ionic motion are typically larger than those related to ferroelectric polarization switching.^28^ It thus follows that ferroelectric hafnium oxide may display the coexistence of ferroelectric switching and ionic motion-driven electroresistance. The benefits of ferroelectric memories compared with those involving ionic motion (i.e., RRAM) is the lower power consumption in addition to improved endurance.^1^ Therefore, distinguishing ionic and ferroelectric-driven effects in ferroelectric tunnel junctions is crucial for applications.

In fact, the dispersion of ER values of junctions of ferroelectric HfO_2_ found in the literature is probably related to the intricate intertwining of ionic and ferroelectric contributions to the measured ER (see Supporting Information S1).^6−20^ Previous works have demonstrated in thick (10 nm) polycrystalline ferroelectric doped HfO_2_ films that after conductive channel formation and subsequent recovery of high resistance state, i.e., conductive channel rupture, the ferroelectric nature of the material is preserved.^29^ However, in the case of ultrathin films, as required for advanced applications,^2^ it remains to be elucidated if, after the formation of conductive channels, ferroelectricity is preserved and tunnel electroresistance resulting from ferroelectric polarization switching still persists. Ferroelectric tunnel junctions of epitaxial films have revealed clear signatures of their ferroelectric character down to 2 nm.^19,30^ On the other hand, epitaxial films allow the fine control of their microstructure, allowing one to tune the relative weight of ferroelectric and ionic contributions to ER.^10,13^ It has also been demonstrated that conducting channels can be blocked by the deposition of appropriate capping layers.^15,31^ Therefore, epitaxial ferroelectric HfO_2_ films offer a unique opportunity to assess the ferroelectric character of films after conducting filament formation (soft breakdown) and to elucidate if ferroelectric polarization switching still leads to an ER, as in the pristine state.

Here, we report on the electroresistance of 4.6 nm epitaxial Hf_0.5_Zr_0.5_O_2_ (HZO) tunnel junctions. Pristine films show a sizable electroresistance (ER ≈ 57%) between high and low resistance states (HRS and LRS, respectively), and ferroelectric characterization shows that electroresistance results from ferroelectric switching. A soft breakdown is induced by the application of a suitable voltage pulse, which leads to a drop in the resistance of the device of about 5 orders of magnitude. Despite the high conductivity, appropriate measurements allow us to identify the signatures of ferroelectric switching and the related ER. In addition, impedance spectroscopy allows us to disclose the nature of the conductive channels in the reported epitaxial devices and to determine the changes that occurred at and under the electrodes.

## Materials and Methods

### Sample Preparation

**Figure 1:**
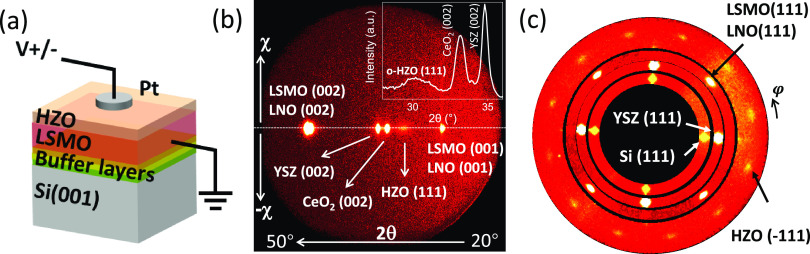
(a) Sketch of the 4.6 nm HZO film on LSMO on buffered Si with Pt top electrodes and its electrical contact configuration. (b) XRD 2θ-χ frame and integrated scan of the sample. Integrated along the χ scan (±5° around χ = 0°), zoom around HZO(111) is also included, corresponding to the θ–2θ scan. (c) XRD pole figure around asymmetric Si, YSZ, LSMO (111), and o-HZO(−111) reflections.

The multilayer (Figure 1a) was fabricated in a single process by pulsed laser deposition (PLD). A complex stack of buffer layers was deposited on Si to achieve subsequent epitaxial growth of HZO. Yttria-stabilized zirconium oxide (YSZ) and CeO_2_ are both grown at the same substrate temperature *T*_s_ = 800 °C and oxygen pressure *P*_O_2__ = 4 × 10^–4^ mbar, and LaNiO_3_ is grown at *T*_s_ = 700 °C and *P*_O2_ = 0.15 mbar. La_2/3_Sr_1/3_MnO_3_ (LSMO), which serves as a template for HZO growth and as a bottom metallic electrode, was deposited at *T*_s_ = 700°C and *P*_O_2__ = 0.1 mbar. HZO was deposited at *T*_s_ = 800°C and *P*_O_2__ = 0.1 mbar at a laser repetition rate of 2 Hz. Epitaxial HZO films on Si (*P*_r_ = 18 μC/cm^2^ for 9 nm film)^32^ show similar polarization to those grown on the SrTiO_3_ perovskite substrate (*P*_r_ = 20 μC/cm^2^ for 9.5 nm film)^33^ using the same PLD conditions. Top Pt electrodes (20 μm of diameter and 20 nm of thickness) were grown ex situ at room temperature using a stencil mask by DC sputtering. More details about growth conditions and structural characterization can be found elsewhere.^32^

### Structural Characterization

The θ–2θ scans were performed using a Siemens D5000 diffractometer. A general area detector diffraction system (GADDS) was used to acquire 2θ-*χ* frames and pole figures around Si, YSZ, LSMO (111), and HZO (−111) asymmetric reflections.

### Electrical Measurement

Electrical measurements were performed by contacting the bottom electrode LSMO and one top Pt electrode using the TFAnalyser2000 platform (aixACCT Systems GmbH). Polarization measurement was done by integrating the current through the time of collected *I*–*V* curves at the indicated frequency. The ER loops were recorded after trapezoidal writing pulses (with amplitude *V*_W_ and duration time τ_W_) and a delay time (τ_D_ = 1 s) by applying a linear *V*_R_(t) triangular pulse of 1 s duration in a specific voltage range (−1.5 to 1.5 V). The resistance value at each *V*_W_ was extracted at *V*_R_ = 1.5 V. The capacitance as a function of frequency was measured by an impedance meter (HP4192LF, Agilent Co.) operated with an oscillation of 300 mV from 10 Hz to 2 MHz with a top–bottom configuration. Equivalent circuit analysis based on the Nyquist plot (*Z*″–*Z*′) was conducted with Zview software^34^ as described in the text.

## Results

The X-ray diffraction (XRD) 2θ–χ frame measured using a two-dimensional (2D) detector (Figure 1b) shows an HZO(111) spot ascribed to the orthorhombic ferroelectric phase. The extracted θ–2θ scan integrated within the χ = ±10° angular range, included in the inset, displays an apparent HZO(111) peak. Note that the peak is broad due to the ultrathin character of the layer. The θ–2θ scan using a point detector (see Supporting Information Figure S2) was used to determine a lattice spacing of ≈ 2.962 Å between HZO(111) planes. The 2θ–χ frame does not show the presence of any other spurious phase, although precedent transmission electron microscopy studies^35^ have identified traces of a minority monoclinic paraelectric phase.^36,37^ Monoclinic, tetragonal, and cubic nonferroelectric phases of ZrO_2_ and HfO_2_ are the stable ones.^4,5^ The ultimate reason for stabilization of the metastable ferroelectric orthorhombic phase is still under debate, and several origins such as doping, capping layers effect,^38^ stress effects,^35,39^ and surface and/or interface energy contributions^40,41^ have been proposed. YSZ(002), CeO_2_(002), and LNO (001) spots from the buffer layer are also visible. The LSMO(001) reflection overlaps with that of the LNO and are not resolved in the 2θ–χ scans, although they are distinguishable in the θ–2θ scan using a point detector (see Supporting Information Figure S2). φ-scan image of YSZ(111), Si(111), LNO/LSMO(111), and HZO(−111) reflections belonging to the different layers is shown in Figure 1c. Note that CeO_2_ peak overlaps with that of Si. The four peaks of YSZ(111) and CeO_2_/Si(111) are clearly visible. The 45° rotation of the peaks of LNO/LSMO(111) with respect to YSZ(111)/CeO_2_/Si(111) indicates that the cell of LNO/LSMO is rotated by 45°. LSMO has a fourfold symmetry, and three HZO domains are formed, as reported elsewhere.^42^ Summarizing, the HZO film is epitaxial and mostly orthorhombic, with a minor contribution of a spurious nonferroelectric monoclinic HZO phase.

**Figure 2:**
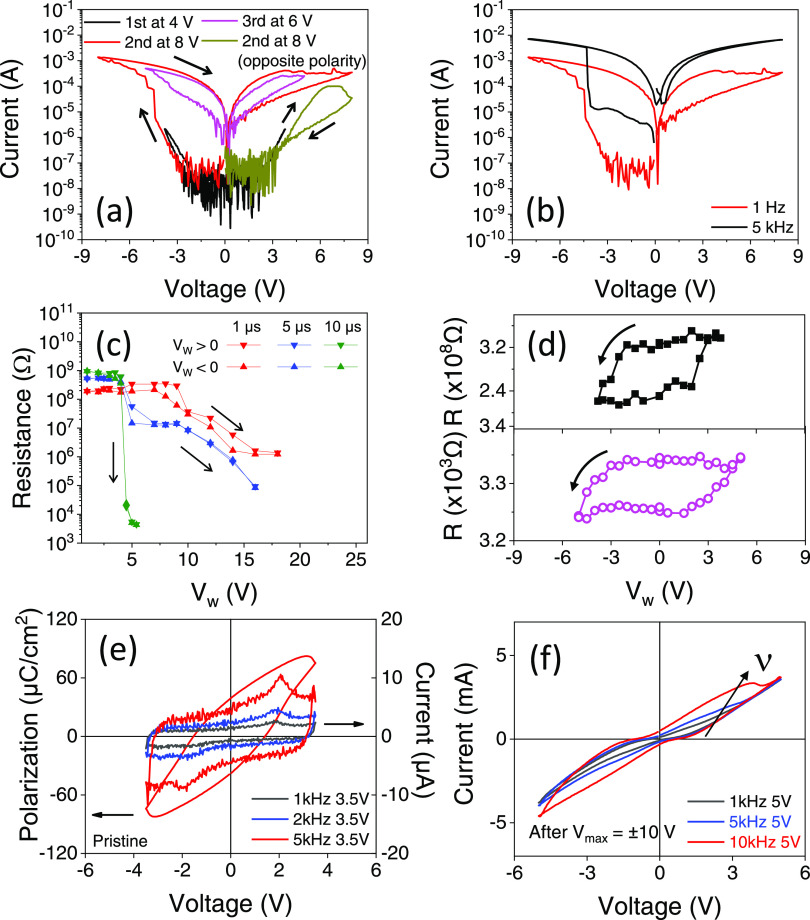
(a) *I*–*V* loops collected before use at indicated voltages in a consecutive manner at 1 Hz. (b) *I*–*V* loops collected at different frequencies: 1 Hz and 5 kHz. (c) *R*(*V*_W_) data of samples with increasing different *V*_w_ pulse amplitudes and indicated τ_w_. (d) ER loop before (top) and after a breakdown (bottom). The ER loops are collected at *V*_max_ = 3.8 and 5 V, respectively (e) *P*–*V* and *I*–*V* loops collected in the pristine state. (f) *I*–*V* loop collected after a breakdown.

Figure 2a shows the *I*–*V* curves collected at 1 Hz up to ±4 V (black), ±8 V (red), and ±6 V (blue) sequentially. It can be observed that during the first loop, the conductivity is small and only hysteresis (negative voltage) is observed. During the second pulse collected up to 8 V, a sudden increase of conductivity is observed near *V*_th_ = −5 V, resembling the conductance increase occurring during the filament formation process in nonferroelectric HfO_2_ films.^23^ If the increasing voltage is applied following the positive polarity path (green line), no abrupt current increase is observed, instead a smaller hysteresis. Finally, the loop collected up to 6 V shows larger conductivity and a clear reversible hysteresis. In Figure 2b, a loop recorded up to 8 V, following the same protocol as for Figure 2a, is shown while measuring at 1 and 5 kHz. Although the measured current in the loop collected at 5 kHz is larger than at 1 kHz due to the displacive current contribution, no significant changes in the hysteretic behavior of the *I*–*V* curves are observed. In Figure 2c, we show the resistance measured after the application of *V*_w_ = −1, +1, −2, +2, −3 V, ···, using the pulses of the indicated duration. It can be observed that for a 10 μs pulse, whose equivalent frequency is 100 kHz, the resistance suddenly decreases at *V*_th_ = ±5 V, which is consistent with the change of conductance observed in Figure 2a,b. A similar change of resistance has been observed earlier and claimed to be due to ionic motion and the concomitant formation of conductive channels.^10^ The sudden change of resistance has also been observed in thinner HZO films (*t* = 2.2, 3.6 nm) (see Supporting Information Figure S3). However, a somewhat larger |*V*_th_| is required in thinner films, probably due to a reduction of defect density by diminishing the HZO thickness. When shorter pulses are applied (5 and 1 μs in Figure 2c), the change of resistance is more gradual and occurs at a larger *V*_th_. Electronic conduction in oxides is of the order of 1 cm^2^/V/s,^43^ corresponding to a time scale of ≈1ps. Thus, the observed dependence of *V*_th_ on the pulse width, which is in the μs time range, is an additional indication of the ionic or defect character of the filament formation, as electronic conduction would be much faster. Next, we explore the ER induced by *V*_W_ with |*V*_W_| <|*V*_th_| before and after a soft breakdown. Figure 2d, top, displays the R(*V*_W_) loop collected in the pristine state (without applying any previous electric stimuli to the junction). It can be observed that the electroresistance loop R(*V*_W_) is reversible, as expected if ER is due to ferroelectric polarization switching. ER = (HRS-LRS)/LRS, where HRS and LRS are the high and low resistance, is ≈ 53%, which is similar to, albeit somewhat smaller than, reported values for similar HZO junctions grown on perovskite substrates, probably due to the different microstructure of the films.^10^Figure 2d, bottom, displays the R(*V*_W_) loops measured after a soft breakdown, as evident in the much reduced resistance of the device. It can be appreciated that the R(*V*_W_) is still reversible, suggesting that, indeed, polarization reversal rules the observed ER, which is sizable, although it has decreased to 3.2%. Comparing the two R(*V*_W_) loops in Figure 2d, it is appreciated that R(*V*_W_) switches from LRS to HRS (and vice versa) at similar voltages (≈2 V), suggesting that the coercive field of the ferroelectric HZO has not been modified by the formation of filamentary channels at the soft breakdown voltage (*V*_th_). The observation that both R(*V*_W_) loops in Figure 2d are similarly shifted toward the left indicates the presence of a ferroelectric imprint field pointing down, as commonly observed in other epitaxial HZO films of a similar structure and morphology and grown under the same conditions and ascribed to the presence of defects,^44^ that also persists after the soft breakdown. Thinner HZO films (*t* = 2.2, 3.6 nm) show similar ER loops before and after the breakdown (see Supporting Information Figure S4).

Before, breakdown stability and good retention of the HRS and LRS states have been reported elsewhere in similar films.^10^ After the breakdown, HRS and LRS are also stable, as shown in Supporting Information S5. Analysis of the *I*–*V* curves at a low voltage shows that these are compatible with the presence of conduction due to tunneling before and after the breakdown, as shown in Supporting Information S6.

The *I*–*V* curves collected in the pristine state (Figure 2e, right axis) show the ferroelectric switching current peak demonstrating the ferroelectric nature of the film, with a background displacive current that naturally increases with measuring frequency. Correspondingly, the *P*–*V* loop determined from the integration of the *I*(*V*(*t*)) data (5 kHz) shows clear hysteretic behavior with well-defined ferroelectric switching with remnant polarization 39 μC/cm^2^ and coercive voltage *V*_C_ ≈ 1.5 V. As the ER loop, the *P*–*V* loop is also shifted toward the left indicating the presence of a downward (toward LSMO) imprint. Note that the remnant polarization value is overestimated due to the leakage contribution visible by the round shape of the loop near the maximum applied voltage.^45^Figure 2f displays the *I*–*V* curve collected after the breakdown. It can be first observed that the junctions are more conducting (notice the different current scales in Figure 2e,f), as expected from the more conducting character of the junction after the breakdown. A remarkable aperture of the *I*–*V* loop is also observed. In Figure 2f, we include *I*–*V* loops collected at increasing frequencies. It can be observed that the mentioned aperture increases with the frequency, reflecting the displacive current contribution and related to the presence of surface charge variation in the capacitor, as observed before the breakdown (Figure 2e). Interestingly, in Figure 2f, it can be appreciated that the *V* > 0 branch of the loop collected at the highest frequency (10 kHz) displays a current peak superimposed to the increasing with *V* current, which indicates ferroelectric switching. The polarization switching peak on the negative *V* < 0 branch of *I*–*V* is not distinguishable due to the large leakage and the presence of an imprint field pointing down. Therefore, transport and ferroelectric characterizations show that ER and ferroelectricity are present before and after the breakdown and indicate that both show similar features indicating their connection.

**Figure 3:**
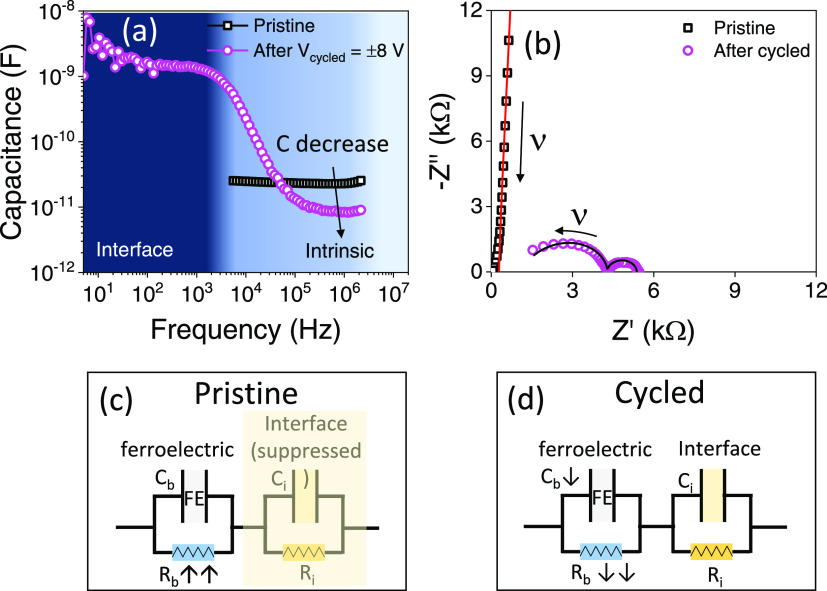
(a) Capacitance as a function of frequency in the pristine and cycled states. (b) Nyquist plot of the impedance of the pristine and after cycling states. Equivalent circuit model for (c) the pristine state when the ferroelectric capacitor is insulating (*R*_b_ ↑↑) and (d) the cycled state when the ferroelectric capacitor becomes leaky (*R*_b_ ↓↓).

To gain further insight into the nature of the conductive channels formed after a soft breakdown, capacitance measurements have been performed. Figure 3a displays the capacitance as a function of frequency (*f*) recorded in the pristine state of the junction. In the accessible experimental range (3 kHz to 3 MHz), the capacitance (≈30 pF) is virtually independent of frequency. Instead, after a breakdown, C(*f*) displays a nonmonotonic variation and two plateaus emerge, signaling the presence of two different contributions to the dielectric permittivity.^46^ Typically, at high frequency, the intrinsic contribution dominates,^47^ which corresponds to the contribution of the bulk of the film. At low frequencies, high capacitance is observed, which is usually ascribed to interface effects.^47^ The interface contribution can result either from electrode/ferroelectric interface layer formation or the presence of grain boundaries.^47,48^ For instance, the formation of a depletion layer at the electrode/ferroelectric interface, necessarily due to its metal/semiconducting nature, results in the formation of an interface capacitance.^49^

Complementary information can be derived from the Nyquist diagrams (Figure 3b) of the impedance before and after the breakdown. The arrows in Figure 3b indicate increasing frequency. Before the breakdown, an almost vertical straight line indicates that the observed response corresponds to a single capacitor. Data before and after the breakdown can be well fitted (line through data points) using a simple model circuit of a series arrangement of two parallel resistance/capacitor systems (*C*_b_,*R*_b_) and (*C*_i_,*R*_i_), where the subindex refers to a bulk (b) and interface (i) contribution as in the Maxwell–Wagner model,^46^ already identified while describing Figure 3a. Here, an additional series resistance term has also been included. Before the breakdown, only a single capacitance contribution is observed (see the sketch in Figure 3c) that we attribute to *C*_b_ with a very large parallel *R*_b_. The *R*_b_ value is beyond the measuring range of the used impedance meter; thus, an accurate value of *R*_b_ cannot be given. Data fit leads to *C*_b_ = 33 pF that corresponds to ε_r_ ≈ 54, using the relation *C* = ε_r_ε_0_*A/t and the nominal (*A*, area and *t*, thickness) geometrical values. After the breakdown, the two contributions become apparent. The bulk one (*C*_b_ = 19 pF and *R*_b_ = 4 kΩ) shows a decrease in both capacitance and parallel resistance compared to that extracted from data of the pristine sample (Figure 3d). The interface contributions lead to *C*_i_ = 48 nF and *R*_i_ = 1 kΩ. See summarized values in Table 1. Note here that the interface contribution is likely to be present before the breakdown, although it is not observed in the impedance spectra due to the predominant bulk contribution. After the breakdown, the ε_r_ extracted from the *C*_i_ capacitance value (48 nF) of the low-frequency contribution is exceedingly large (7.8 × 10^4^), evidencing its extrinsic origin. For the high-frequency contribution, *C*_b_ = 19 pF, which would correspond to ε_r_ ≈ 3,1, is obtained. Note that similar *C*(*f*) data trends have been observed in samples with different HZO thicknesses (*t* = 3.6, 2.2 nm), as shown in Supporting Information Figure S7 and summarized in Table 1.

**Table 1:** Summary of the Fitted Parameters from the Impedance Spectrum of the Samples of the Indicated Thickness Extracted from the Plots Shown in Figures 3b and S7^a^

sample thickness (nm)	stage	*R*_i_ (Ω)	error (Ω)	*C*_i_ (nF)	error (nF)	*R*_b_ (Ω)	error (Ω)	*C*_b_ (pF)	error (pF)	*t*_model_ (nm)	ε_r_
4.6	pristine							32.4	0.18	4.6	53 (fitted)
	cycled	1090	23.3	47.8	2.4	4053	12.5	19.5	0.18	7.7	53 (fixed)
3.6	pristine							39	1	3.6	50.5(fitted)
	cycled	1181	34.7	98.2	6.1	3820	15.9	32	0.32	4.39	50.5 (fixed)
2.2	pristine							40.4	0.53	2.2	32 (fitted)
	cycled	1035	22.6	39.5	2.2	3753	13.5	32.9	0.1	2.7	32 (fixed)

a Note that in the pristine state, the *t*_model_ is fixed and the ε_r_ fitted, whereas in the cycled state, the *t*_model_ is fitted and ε_r_ fixed, according to the value extracted from the fitting of the impedance spectra in the pristine state.

**Figure 4:**
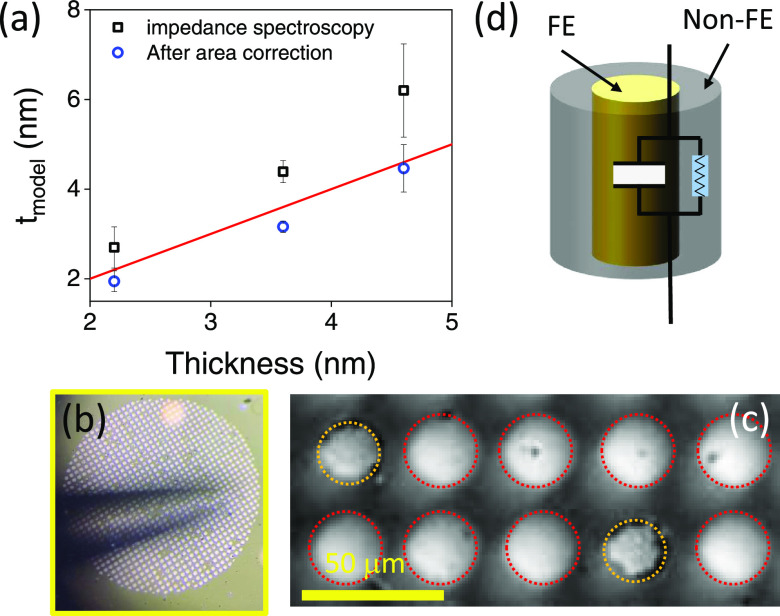
(a) Thickness extracted from the data fitting using the procedure indicated in the text vs the nominal thickness of different samples before and after correcting the device using the area extracted from broken devices shown in panel (c). Error bars correspond to the standard deviation of data collected in three different junctions. (b) Wide-view of the Pt electrodes. (c) Zoom of the Pt top electrodes. The broken devices are in yellow. (d) Sketch of the two ferroelectric and nonferroelectric contributions extracted from capacitance measurements analysis.

The reduction from ε_r_ ≈ 54 to 31 of the bulk contribution to the permittivity of the material after the breakdown can result from the device heating due to the injected current leading to subsequent orthorhombic–monoclinic HZO phase change. This phase transformation can lead to a reduction of the dielectric permittivity,^50^ as experimentally observed here. However, XRD temperature experiments of similar films with similar composition have shown that the orthorhombic phase is stable at least up to 1000 C,^51^ which is much larger than the evaluated temperature increase in similar devices.^52^ Therefore, an intrinsic permittivity decrease related to a phase transformation is unlikely to occur. Alternatively, if the permittivity after the breakdown is fixed to its value before the breakdown, then, assuming the same contact area A, the effective thickness of the capacitor would be increased up to ≈ 7.7 nm. A similar thickness increase is obtained from the *C*_b_ contribution derived from *C*(*f*) of thinner samples, as shown in Figure 4a and summarized in Table 1, where the dependence of the extracted thickness from data fitting is plotted for samples of different thicknesses. However, such a dramatic thickness increase after the breakdown does not appear to be well grounded. Therefore, data suggest that after the breakdown, most likely, the actual area of the capacitor has been modified; more precisely, reduced to account for the observed reduction of *C*_b_. Figure 4b shows a wide-view optical microscopy image of several top Pt electrodes on the sample. The zoom included in Figure 4c shows devices (encircled in yellow) after the breakdown. It can be observed that, at their borders, a darker region appears, which we ascribe to the formation of a higher conductivity region under electrode edges, suggesting that the effective area of the dielectric is indeed reduced. Similar behavior has been observed in other ferroelectric capacitors.^53^ Consistently, according to the piezoresponse force microscopy (PFM) images (see Supporting Information Figure S8), the piezoelectric response at the electrode borders is weaker than at its center. To sum up, the sketch of the device after the breakdown is shown in Figure 4d. It illustrates that, upon a soft breakdown, the ferroelectric intrinsic contribution is preserved, while a conductive region (nonferroelectric) develops near the electrode borders, probably resulting from the higher electric field at electrode edges. In Figure 4a, the thickness extracted considering the device area after the breakdown, evaluated as the brighter region in the broken devices of Figure 4d, is shown as a function of the nominal thickness for three different samples. It is observed that the thickness obtained after the correction is in good agreement with the expected value (red line). Therefore, it can be concluded that the formation of a conductive ring and the concomitant reduction of the effective area of the ferroelectric result in a decrease of the overall resistance and a decrease of the device capacitance, while the dielectric properties of the unperturbed area remain robustly insulating.

## Conclusions

In conclusion, the *I*–*V* curves and electroresistance data of HZO capacitors indicate that a soft breakdown occurs. Data indicate that ferroelectric polarization-related electroresistance exists before the breakdown and persists after the breakdown. Besides, a significant reduction of device capacitance is observed after the breakdown. Analysis of the frequency-dependent capacitance before and after the breakdown allows us to conclude that the formation of a conductive channel results in a reduction of the functional effective area and accounts for both the reduction of capacitance and the reduction of its parallel resistance component. Further characterization including optic and PFM response indicates that the conductive channel forms a ring at the edge of the electrodes. The performed characterization should contribute to the further understanding of the coexisting contributions in ferroelectric HfO_2_ tunneling or other devices and also to their better design. In particular, we have demonstrated that capacitance characterization can help to disclose the presence of extrinsic effects. In addition, the observed modulation of the overall resistance level can be useful for developing more optimal device engineering strategies.

## Supporting Information Available

Comparison with literature values and structural, electric, and PFM characterization (PDF)
