# Supplementary material for: Ferroelectric Electroresistance after a Breakdown in Epitaxial Hf0.5Zr0.5O2 Tunnel Junctions
Source: ACS Appl Electron Mater. 2023 Jan 30;5(2):740–7. doi: 10.1021/acsaelm.2c01186 (PMC9979785; doi:10.1021/acsaelm.2c01186)
Supplement: Supplementary file 1 — el2c01186_si_001.pdf [file el2c01186_si_001.pdf]

Supporting Information

Ferroelectric Electroresistance After Breakdown in Epitaxial $Hf_{0.5}Zr_{0.5}O_2$ Tunnel Junctions

Xiao Long,[1] *Huan Tan,*[1] *Florencio Sánchez,*[1] *Ignasi Fina,*[1,*] *Josep Fontcuberta,*[1,*]

[1]Institut de Ciència de Materiales de Barcelona (ICMAB-CSIC), Campus UAB, Bellaterra 08193, Catalonia, Spain

*ifina@icmab.es; fontcuberta@icmab.cat

Supporting Information S1

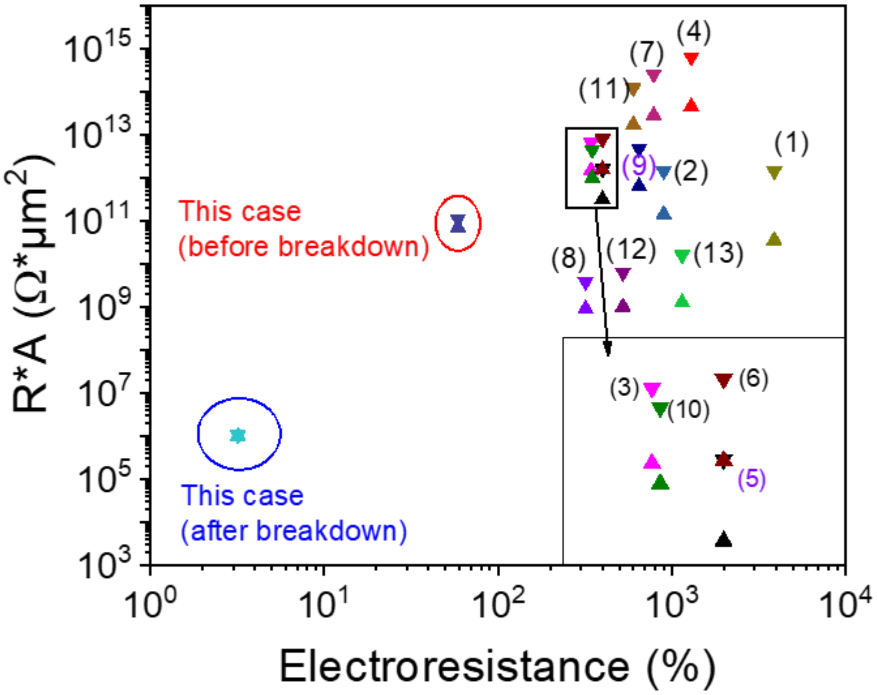

Figure S1.Survey of literature[1-13] values of resistance normalized to the area versus electroresistance in junctions based on ferroelectric doped HfO_2. Results presented in the present manuscript before and after breakdown are also included.

Supporting Information S2

Figure S2 shows XRD θ-2θ scans of the as-prepared stack of Si(001)/buffer layer/LSMO/HZO. The pattern shows (00*l*) reflections corresponding to the Si substrate and the LSMO, LNO, CeO_2 and YSZ layers. o-HZO (111) peak position is around 2θ ≈ 30.14° (see the zoomed panel on the right side), indicating that the inter-planar space of $d_{o\text{-}HZO(111)} \approx 2.962$Å. Notice also that LSMO (00*l*) overlaps with one of the buffer layer LNO (00*l*).

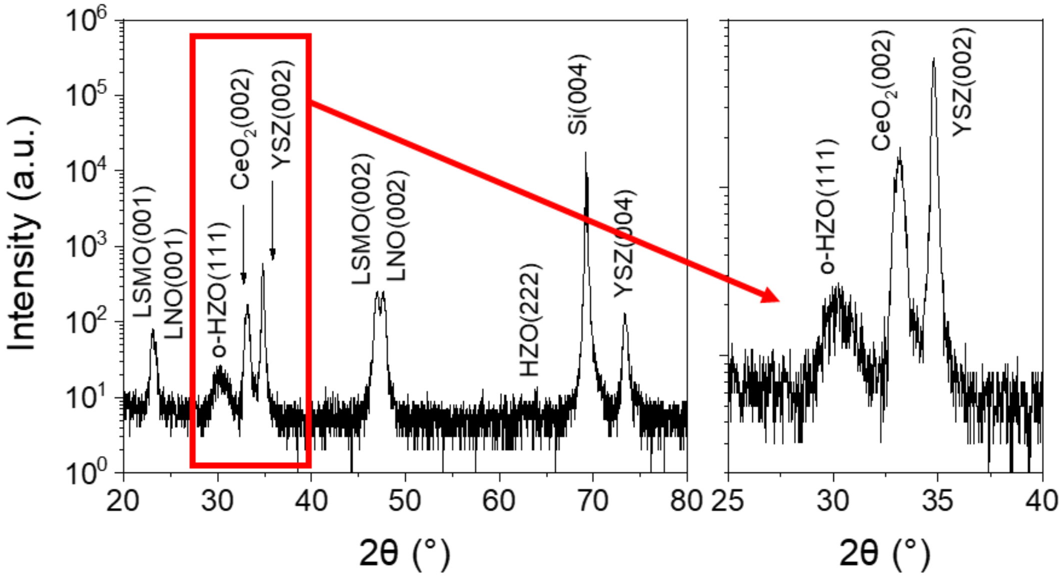

Figure S2. XRD θ-2θ scans of a Si(001)/buffer layer/LSMO/HZO stack in range of 2θ = 20°~80°, with a zoomed region around the HZO (111) (2θ = 25 ° ~ 40°).

Supporting Information S3

Resistance data was recorded as a function of writing voltage respectively for HRS and LRS for samples with different HZO thickness (Figure S3). This measurement was conducted by prepoling samples following a sequence: V_w = ±1 V, ±2 V, ± 2.5V, etc. For samples of t = 4.6nm and t = 3.6nm, this V_{th} is roughly around ± 4.5V; however, for the thinnest sample t = 2.2nm, V_{th} ≈ ±7V. The tendency of increasing threshold voltage with HZO thickness is consistent with the information shown in Figure S3.

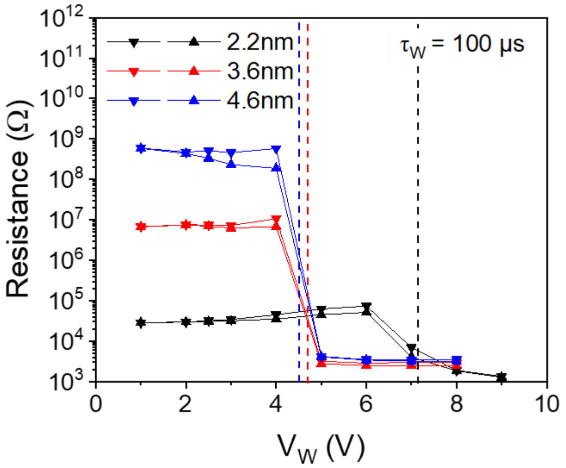

Figure S3. R(V_W) data of samples with different HZO thickness (t = 2.2, 3.6, 4.6 nm). The down triangle symbol (▽) represents positive prepoling while the up triangle symbol (△) represents negative prepoling. The writing time for all prepoling are the same τ_w = 100 μs.

Supporting Information S4

R(V_w) loops before and after the soft-breakdown are shown for samples of t = 3.6 nm and 2.2nm in Figure S4(a,b), respectively. The ER in the sample t = 3.6nm changes from ER = 26% to ER = 9.8% [Figure S4(a)], while in sample t = 2.2nm it changes from ER = 33% to ER = 1.6% [Figure S4(b)].

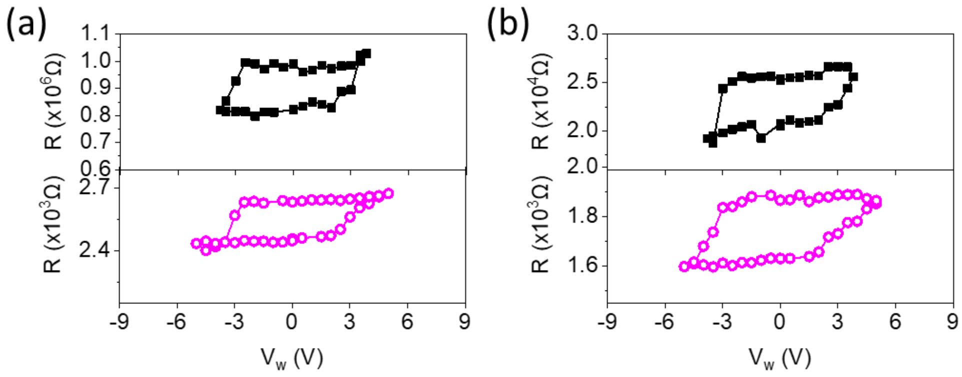

Figure S4. R-V loops collected up to V_{max} = 3.8V, 8V and 5V sequentially in samples with different HZO thickness (a) 3.6 nm and (b) 2.2 nm. And zoomed loops of the 1st (top) and 3rd (bottom) collected respectively for two samples (a) 3.6 nm and (b) 2.2 nm.

Supporting Information S5

The HRS and LRS states dependence on τ_w is shown in Figure S5(a). It can be observed that for V_w of 5 V the HRS and LRS are well-defined and stable as expected from the fact that these result from different ferroelectric polarization states as discussed in the main text. This is ascribed to the presence of ionic mechanisms involved on the resistance drop. For V_w = 8 V, the HRS and LRS states start to open above 100 µs, highlighted as the slow region. This aperture is most probably related to the ionic motion, which is expected to be more prominent while applying longer pulses as already observed in other ferroelectric films. Regarding endurance experiments for V_w = 5 and 8 V and fix τ_w = 100 µs, the resistance contrasts up to 200 cycles is stable [Figure S5(b)], although for V_w = 8 V the variability is larger.

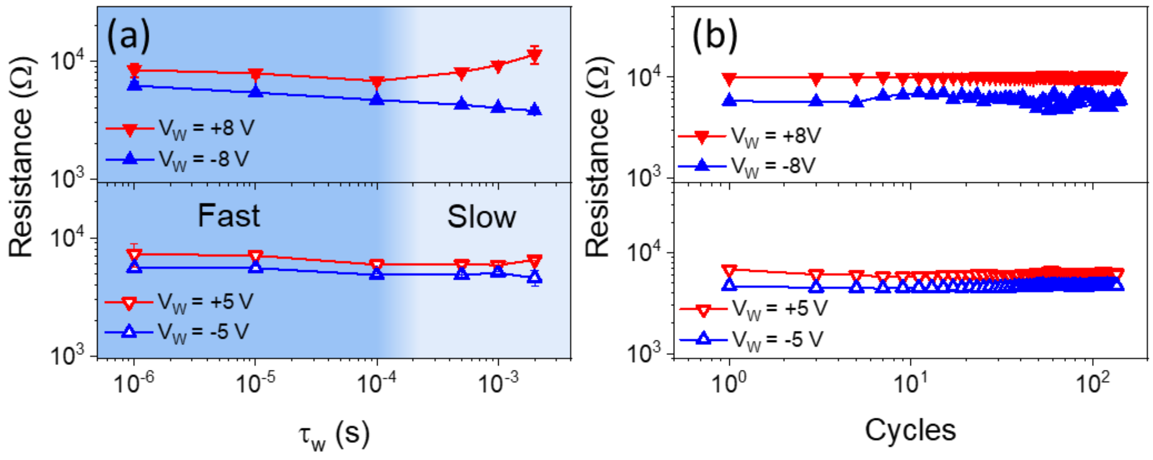

Figure S5. (a) Resistance after application of V_w of indicated amplitude versus τ_w. (b) Resistance after application of V_w of indicated amplitude versus cycles of amplitude V_w, τ_w = 100 µs in the collected data of a and c.

Supporting Information S6

We now try to disclose the conduction mechanisms in the explored system. Figure S6(a) shows the dependence of the resistance states versus thickness. The observed exponential dependence suggests tunneling current as a possible conductance mechanism. After the breakdown, although final values of resistance show some dispersion accounted by the error bar of the plot, which has been calculated by the standard deviation of the collected resistance values, a similar exponential dependence is observed. Similar conclusion can be extracted from the I-

V characteristics analysis. In Figure S6(b), the I-V curves after prepoling the sample with + or – 5 V (as indicated) are shown. It can be observed that the curves show sigmoidal shape. Lines through data points correspond to the Brinkman model fitting.[14] The obtained parameters are summarized in see Supporting Information Table S1, which show reasonable agreement with the literature.[5, 9, 15] In Figure S6(c), the I-V curves after prepoling the sample with + or – 5 V after breakdown are shown. It can be observed that the contrast between both curves is smaller in agreement with the observed smaller ER. After breakdown, it can be observed larger conductivity, which indicates the presence of a metallic-like channel. In fact, Brinkman model fitting results in smaller barrier height and thickness, as expected by the fact that the conductive channel would cause an extrinsic reduction of these parameters (Table S1). The obtained parameters from data fitting are nearer to the expected ones, if a parallel resistance is considered (Table S1).

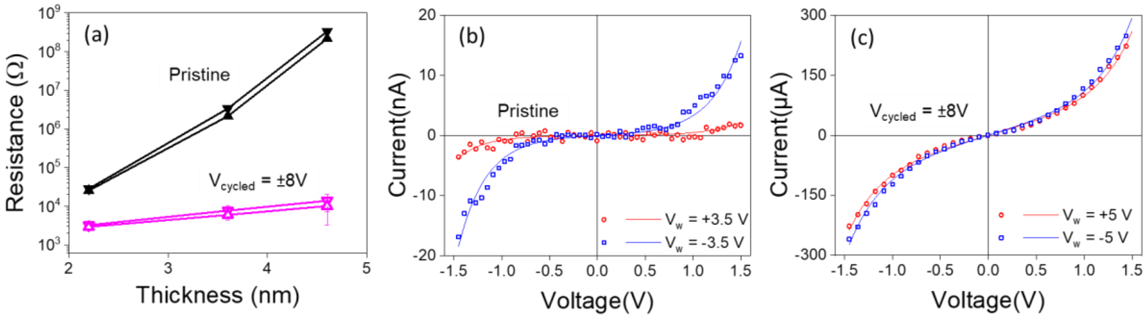

Figure S6. (a,b) I-V curves collected respectively after writing with Vw = +3.5 V and Vw = −3.5 V as indicated for the (b) pristine state, and Vw = +5 V and Vw = −5 V for (c) after Vcycled = ±8 V. (c) Dependence of ON and OFF resistance states on the HZO thickness for pristine and after Vcycled = ±8 V.

Table S1. Barrier parameters extracted from fittings are shown in Figure. 4 (b)(c), including effective thickness (t_{eff}), barrier height for both interfaces (Φ_{LSMO} and Φ_{Pt}), and the fitting quality chi^2. Exclusively for status after breakdown, both conventional Brinkman model and an improved Brinkman model with parallel resistance (fixed R = 18kΩ).

Break	State	Φ_{LSMO} (eV)	Φ_{Pt} (eV)	t_{eff} (nm)	chi2
Before	LRS -3.5 V	2.06	2.85	4.6	9.51×10^{-9}
Before	HRS +3.5 V	1.76	3.44	4.7	5.38×10^{-9}
After	LRS -5 V	2.49	3.04	2.79	5.05×10^{-5}
After	HRS +5 V	2.40	3.07	2.82	6.80×10^{-5}
After (Parallel R=18 kΩ)	HRS +5 V	1.93	1.97	3.5	6.1×10^{-5}
After (Parallel R=18 kΩ)	LRS -5 V	1.96	2.06	3.4	7.22×10^{-5}

Supporting Information S7

Capacitance (*C*-*f*) and impedance (*Z''*-*Z'*) measurement conducted in sample with different HZO thickness. Similar behavior is observed for all samples irrespectively of their thickness.

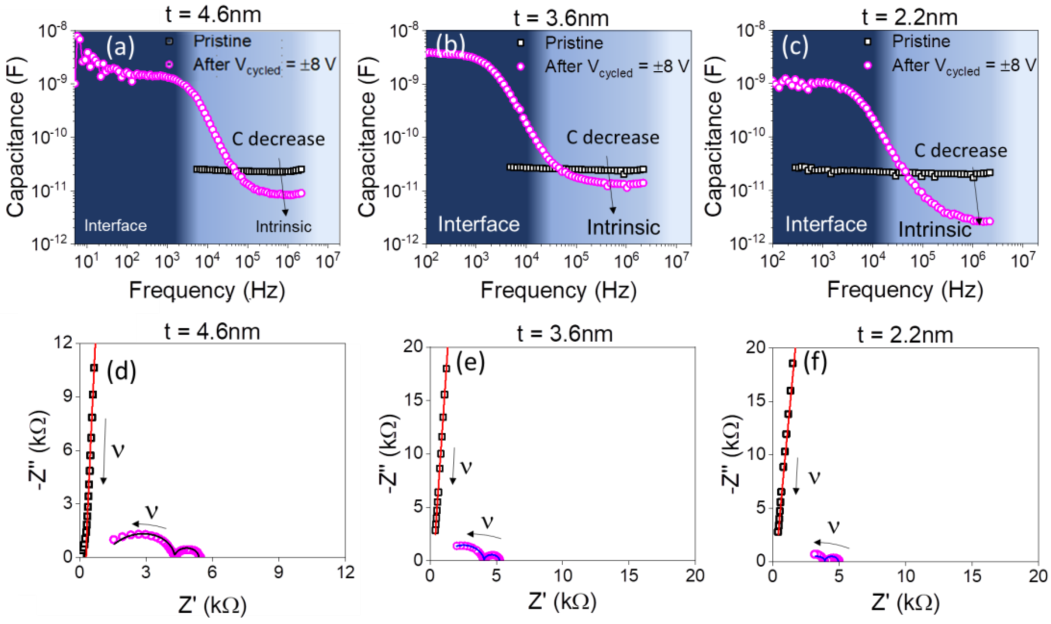

Figure S7. Capacitance as a function of frequency in pristine and cycled states for samples with different HZO thickness (a) t = 4.6nm, (b) t = 3.6nm, (c) t = 2.2nm. And each corresponding Nyquist plot (d) t = 4.6nm, (e) t = 3.6nm, (f) t = 2.2nm.

Supporting Information S8

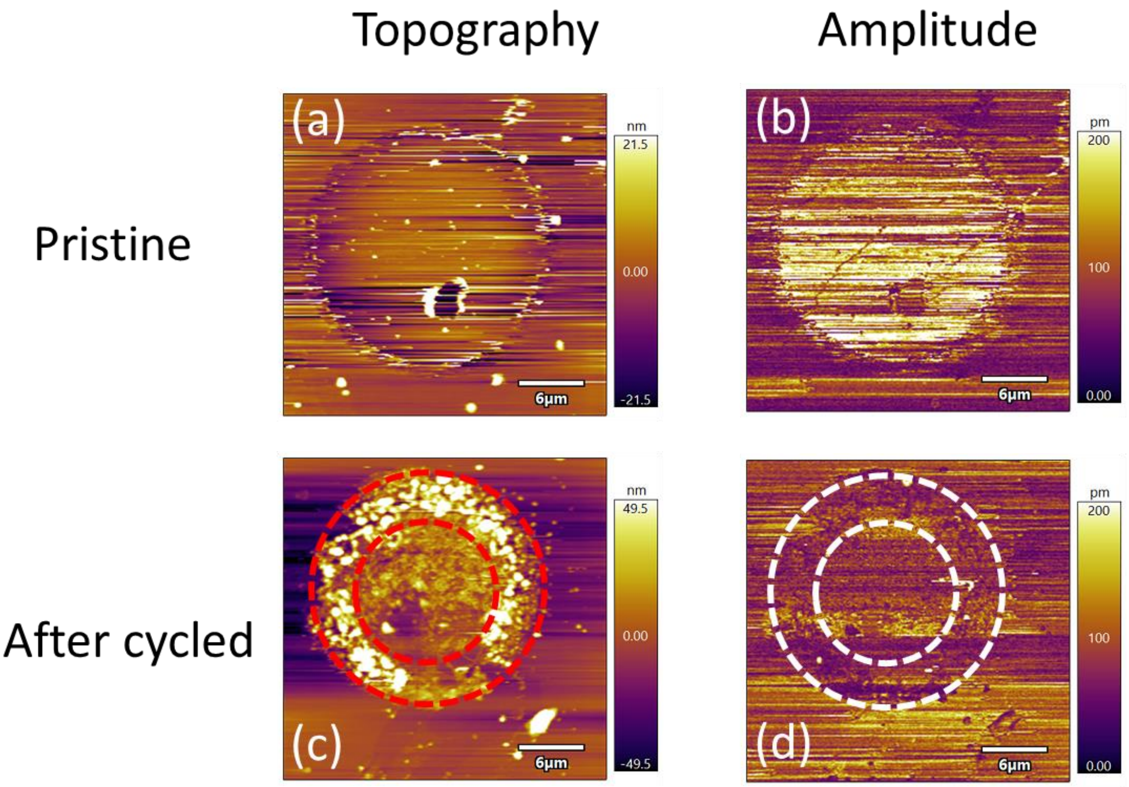

Figure S8. PFM topography on Pt electrode region for (a) pristine and (c) after cycled. PFM amplitude on Pt electrode region for（b）pristine and (d) after cycled. For Pt electrode after cycled, a ringed wrinkle was observed from its topography, as shown in the region between two dashed circles in (c), which indicates to a volume expansion caused by filament formation. And the piezo response in this area is considerably lower as shown in its amplitude (d).

Supporting Information References

(1) Ambriz-Vargas, F.; Kolhatkar, G.; Broyer, M.; Hadj-Youssef, A.; Nouar, R.; Sarkissian, A.; Thomas, R.; Gomez-Yáñez, C.; Gauthier, M. A.; Ruediger, A. A Complementary Metal Oxide Semiconductor Process-Compatible Ferroelectric Tunnel Junction. *ACS Appl. Mater. Interf.* **2017,** *9* (15), 13262-13268.
(2) Ambriz-Vargas, F.; Kolhatkar, G.; Thomas, R.; Nouar, R.; Sarkissian, A.; Gomez-Yáñez, C.; Gauthier, M.; Ruediger, A. Tunneling Electroresistance Effect in a Pt/$Hf_{0.5}Zr_{0.5}O_2$/Pt Structure. *Appl. Phys. Lett.* **2017,** *110* (9), 093106.
(3) Yoong, H. Y.; Wu, H.; Zhao, J.; Wang, H.; Guo, R.; Xiao, J.; Zhang, B.; Yang, P.; Pennycook, S. J.; Deng, N. Epitaxial Ferroelectric $Hf_{0.5}Zr_{0.5}O_2$ Thin Films and Their Implementations in Memristors for Brain-Inspired Computing. *Adv. Funct. Mater.* **2018,** *28* (50), 1806037.

(4) Max, B.; Hoffmann, M.; Slesazeck, S.; Mikolajick, T. In *Ferroelectric Tunnel Junctions Based on Ferroelectric-Dielectric $Hf_{0.5}Zr_{0.5}O_2/Al_2O_3$ Capacitor Stacks*, 2018 48th European Solid-State Device Research Conference (ESSDERC), IEEE: 2018; pp 142-145.
(5) Sulzbach, M. C.; Estandía, S.; Long, X.; Lyu, J.; Dix, N.; Gàzquez, J.; Chisholm, M. F.; Sánchez, F.; Fina, I.; Fontcuberta, J. Unraveling Ferroelectric Polarization and Ionic Contributions to Electroresistance in Epitaxial $Hf_{0.5}Zr_{0.5}O_2$ Tunnel Junctions. *Adv. Electron. Mater.* **2019,** *6*, 1900852.
(6) Ryu, H.; Wu, H.; Rao, F.; Zhu, W. Ferroelectric Tunneling Junctions Based on Aluminum Oxide/ Zirconium-Doped Hafnium Oxide for Neuromorphic Computing. *Sci. Rep.* **2019,** *9* (1), 20383.
(7) Max, B.; Hoffmann, M.; Slesazeck, S.; Mikolajick, T. Direct Correlation of Ferroelectric Properties and Memory Characteristics in Ferroelectric Tunnel Junctions. *IEEE Journal of the Electron Devices Society* **2019,** *7*, 1175-1181.
(8) Wei, Y.; Matzen, S.; Quinteros, C. P.; Maroutian, T.; Agnus, G.; Lecoeur, P.; Noheda, B. Magneto-Ionic Control of Spin Polarization in Multiferroic Tunnel Junctions. *npj Quantum Mater.* **2019,** *4* (1), 1-6.
(9) Sulzbach, M. C.; Estandia, S.; Gazquez, J.; Sánchez, F.; Fina, I.; Fontcuberta, J. Blocking of Conducting Channels Widens Window for Ferroelectric Resistive Switching in Interface-Engineered $Hf_{0.5}Zr_{0.5}O_2$ Tunnel Devices. *Adv. Funct. Mater.* **2020,** *30* (32), 2002638.
(10) Goh, Y.; Hwang, J.; Jeon, S. Excellent Reliability and High-Speed Antiferroelectric $Hfzro_2$ Tunnel Junction by a High-Pressure Annealing Process and Built-in Bias Engineering. *ACS Appl. Mater. Interf.* **2020,** *12* (51), 57539–57546.
(11) Shekhawat, A.; Walters, G.; Yang, N.; Guo, J.; Nishida, T.; Moghaddam, S. Data Retention and Low Voltage Operation of $Al_2O_3/Hf_{0.5}Zr_{0.5}O_2$ Based Ferroelectric Tunnel Junctions. *Nanotechnology* **2020,** *31* (39), 39LT01.
(12) Prasad, B.; Thakare, V.; Kalitsov, A.; Zhang, Z.; Terris, B.; Ramesh, R. Large Tunnel Electroresistance with Ultrathin $Hf_{0.5}Zr_{0.5}O_2$ Ferroelectric Tunnel Barriers. *Adv. Electron. Mater.* **2021**, 2001074.
(13) Yu, T.; He, F.; Zhao, J.; Zhou, Z.; Chang, J.; Chen, J.; Yan, X. $Hf_{0.5}Zr_{0.5}O_2$-Based Ferroelectric Memristor with Multilevel Storage Potential and Artificial Synaptic Plasticity. *Science China Materials* **2021,** *64* (3), 727-738.
(14) Gruverman, A.; Wu, D.; Lu, H.; Wang, Y.; Jang, H.; Folkman, C.; Zhuravlev, M. Y.; Felker, D.; Rzchowski, M.; Eom, C.-B. Tunneling Electroresistance Effect in Ferroelectric Tunnel Junctions at the Nanoscale. *Nano Lett.* **2009,** *9* (10), 3539-3543.
(15) Cervo Sulzbach, M.; Tan, H.; Estandía, S.; Gàzquez, J.; Sánchez, F.; Fina, I.; Fontcuberta, J. Polarization and Resistive Switching in Epitaxial 2 nm $Hf_{0.5}Zr_{0.5}O_2$ Tunnel Junctions. *ACS Appl. Electron. Mater.* **2021,** *3* (8), 3657-3666.
